# Effects of Core Stability Training on Balance, Standing, and Gait in Children with Mild Cerebral Palsy: A Randomized Controlled Trial

**DOI:** 10.3390/healthcare13111296

**Published:** 2025-05-29

**Authors:** Nancy Mohamed, Mohamed B. Ibrahim, Osama A. El-Agamy, Monira I. Aldhahi, Sara Y. Elsebahy

**Affiliations:** 1 Department of Physical Therapy for Pediatrics, Kafr El-Sheikh University, Kafr El Sheikh 33516, Egypt; nancymohamed054@gmail.com (N.M.); mbedair302@gmail.com (M.B.I.); sarah_elsehy2014@pt.kfs.edu.eg (S.Y.E.); 2 Faculty of Medicine, Kafrelsheikh University, Kafr El Sheikh 33516, Egypt; osamaalagmy2@gmail.com; 3 Department of Rehabilitation Sciences, College of Health and Rehabilitation Sciences, Princess Nourah bint Abdulrahman University, P.O. Box 84428, Riyadh 11671, Saudi Arabia

**Keywords:** diplegic cerebral palsy, gait, balance, gross motor function, core stability exercises

## Abstract

**Background/Objectives**: Children with diplegic spastic cerebral palsy (CP) often present with impaired postural control, poor balance, and gait abnormalities that negatively affect their functional mobility and independence. Core stability, which is the ability to control the position and movement of the trunk, is considered a critical component in maintaining postural alignment and improving gross motor function. This study aimed to investigate the impact of a structured core stability exercise program on the standing ability, functional balance, and gait parameters of children diagnosed with diplegic spastic CP. **Methods**: Forty children (28 males, 12 females) aged 4–10 years with a clinical diagnosis of diplegic spastic cerebral palsy were randomly allocated into two groups (*n* = 20 each). The study group underwent a 12-week core stability exercise program in addition to a standardized physiotherapy regimen, which was conducted three times per week. The control group received the physiotherapy program alone. Functional outcomes were assessed pre- and post-intervention using the Gross Motor Function Classification System (GMFCS), Pediatric Balance Scale, and Kinovea software for gait analysis. **Results**: Both groups demonstrated statistically significant improvements in all measured variables after the intervention. However, the study group showed significantly greater improvements in standing ability (9%), balance (9%), and gait parameters (*p* < 0.05), particularly in knee flexion, ankle dorsiflexion, and plantar flexion, during gait cycles. **Conclusions**: Core stability training resulted in superior enhancements in balance, standing, and gait performance compared with physiotherapy alone in children with diplegic spastic cerebral palsy.

## 1. Introduction

Cerebral palsy (CP) is a group of non-progressive neurological disorders resulting from damage to the developing brain that primarily affects posture, movement, and functional activity in infants and children [1]. Children with CP often experience significant limitations in performing basic motor tasks, such as running, stair climbing, walking on uneven surfaces, and achieving independent ambulation. These challenges stem from impaired motor control, spasticity, and musculoskeletal dysfunctions. As a result, the primary therapeutic goals for this population focus on improving functional mobility, particularly walking, and enhancing the capacity to perform activities of daily living [2,3].

Children with CP exhibit varying degrees of functional impairment [4]. A prominent clinical subtype of CP is spastic diplegia, characterized by hypertonia in the lower extremities, significant trunk weakness, reduced postural control [5], loss of axial control, and impaired intersegmental coordination, resulting in a broad range of functional limitations [6]. Postural control can maintain balance, stability, and orientation during movement [7]. Deficits in balance control are closely linked to impaired gait, as stability is fundamental to the execution of all motor actions [8]. Postural systems, including proximal stability, antigravity mechanisms, righting and equilibrium reactions, and postural fixation strategies, are affected in children with CP [9].

Core stability training has been increasingly recommended as a therapeutic strategy to improve postural control and movement efficiency in individuals with neuromotor impairment. These exercises target the shoulder girdle and abdominal, pelvic, and spinal muscles, which are essential for providing a stable base from which the limbs can operate effectively while maintaining postural alignment [10]. Core stability encompasses components such as muscle strength, balance, endurance, and neuromuscular control of the trunk and pelvis [11,12]. The core musculature functions as an integrated unit to stabilize the body during both static and dynamic tasks such as maintaining upright standing or coordinating limb movements [13].

In addition to supporting posture and movement, core stability plays a crucial role in minimizing the risk of injury and addressing muscular imbalance. It contributes to improved functional coordination and balance of both the upper and lower extremities by enhancing neuromuscular control across the kinetic chain [14]. Core stability exercises have been shown to facilitate physical performance by improving lumbar spine stabilization and activating the deep abdominal musculature, which are essential for maintaining trunk integrity during movement [15].

The lumbo-pelvic region serves as the foundation for both load-bearing and functional movements, offering protection to the spinal cord and nerve roots while supporting the efficient transfer of forces to and from the upper and lower limbs [16]. During dynamic actions, such as running, jumping, and throwing, the core musculature is vital for stabilizing the trunk and maintaining spinal alignment [17]. Effective postural and balance control is considered a prerequisite for functional ambulation and safe gait performance [18]. Consequently, core stability training is widely recommended to improve dynamic balance, trunk control, and gait efficiency, particularly in populations with neurological impairments [19]. Therefore, this study aimed to examine the effects of core stabilization exercises on gait performance, functional balance, and standing ability in children with spastic diplegic cerebral palsy.

## 2. Materials and Methods

### 2.1. Study Design and Participants

This study employed a randomized controlled trial (RCT) design and was registered on ClinicalTrials.gov (registration number: NCT06473181; date: 24 June 2024). Both males and females diagnosed with spastic diplegic cerebral palsy, aged between 4 and 10 years, were recruited from the Pediatric Physical Therapy Clinic at Kafr El-Sheikh University. The participants were randomly assigned to two equal groups (*n* = 20 per group).

Inclusion criteria required participants to have a spasticity grade of 1 or 1+ in the lower limbs as assessed by the Modified Ashworth Scale (MAS) and to be classified within level I or II of the Gross Motor Function Classification System (GMFCS), so that children can stand and walk on their own. Additionally, participants were required to have sufficient cognitive and communication abilities to follow verbal instructions for both treatment and assessment procedures. Children were excluded if they (1) had undergone recent lower limb orthopedic surgery; (2) had significant limb deformities; (3) had severe visual, auditory, or perceptual impairments; (4) received botulinum toxin injections in the lower limbs within the previous six months; or (5) had a history of epileptic seizures, as some of these factors can make the movement of the lower limbs difficult, and others can impair the child’s cognition and communication. The sample size was calculated using Cochran’s formula with a confidence level of 95% (Z = 1.96), estimated proportion (*p* = 0.35), and margin of error (e = 0.148). The minimum sample size required was determined to be 40 participants, with 20 children allocated to each group [20].

### 2.2. Study Design and Randomization

This study was conducted as a randomized controlled trial (RCT) between June and October 2024. Randomization was performed using Microsoft Excel. Patient names are listed in one column and random numbers are generated using the RAND function in the second column. Based on the ascending order of these random numbers, the participants were randomly assigned to either the study or control group.

### 2.3. Outcome Measures

#### 2.3.1. Pediatric Balance Scale (PBS)

A modified version of the Berg Balance Scale, the PBS consists of 14 functional balance tasks scored from 0 to 4, yielding a total score out of 56. It is specifically designed for children with mild to moderate motor disabilities and has demonstrated high inter-rater and test–retest reliability [21], as well as excellent face and content validity in children with spastic cerebral palsy [22].

#### 2.3.2. Kinovea Motion Analysis Software

Kinovea is a 2D open-source software used to assess joint angles and motion during gait (Kinovea version 0.9.5; https://www.kinovea.org/). Three colored markers were placed on the greater trochanter, lateral femoral condyle, and lateral malleolus. The knee angle was calculated between the lines connecting the greater trochanter to the femoral condyle, and the femoral condyle to the lateral malleolus. The ankle angle was determined by drawing lines between the second metatarsal head and calcaneus, and from the femoral condyle to the lateral malleolus [23,24,25,26]. Kinovea has been validated for use in gait and movement analysis, showing strong intra- and inter-rater reliability [24].

#### 2.3.3. Gross Motor Function Measure (GMFM)

The GMFM-88 was used to evaluate gross motor function, focusing on the standing dimension (Dimension D). Each item is scored on a four-point ordinal scale, and children were allowed up to three attempts per item. If the item was successfully performed on the first attempt, no further attempts were required. The overall score is expressed as a percentage based on the total score for that dimension [27].

### 2.4. Treatment Procedures

All interventions were conducted at the Pediatric Physical Therapy Outpatient Clinic over a 12-week period. Three licensed physical therapists participated in implementing the intervention protocol. Prior to the study, participants received standardized training to ensure consistency in treatment delivery. Each therapist was responsible for a subset of the participants and followed the same intervention procedures under the supervision of the principal investigator.

Participants in the study group received individualized treatment consisting of three sessions per week, each lasting one hour. The first 30 min of each session comprised a conventional physiotherapy program, which was also administered to the control group. The remaining 30 min were dedicated to a structured core stability training program delivered in a progressive format across the three levels of difficulty (Table 1). Beginner level (level 1) included exercises such as belly draw-in (20 repetitions), belly draw-in combined with double knee-to-chest (10–20 repetitions), and supine trunk twists (10–20 repetitions). The intermediate level (level 2) introduced supine trunk bridging (3–5 repetitions) and twisting with a medicine ball (10–20 repetitions). The advanced level (level 3) consisted of trunk bridging with the head supported on a physio-ball (held for 3–5 s and repeated 10–20 times) and prone bridging (3–5 repetitions). Each exercise was followed by a rest period of 30 to 60 s, and the full sequence of exercises was repeated three to five times based on the child’s tolerance and progression [28].

Participants in the control group received 30 min of conventional physiotherapy, which included postural control exercises in various positions, such as kneeling, stepping, and single-leg stance. A series of stretching exercises targeting the hip flexors, adductors, knee flexors and extensors, and calf muscles were also performed (each stretch was held for 20 s and repeated three to five times). The strengthening component of the program focused on key lower limb and core muscle groups, including hip abductors (trained in side-lying position), gluteal muscles (prone hip extension), hamstrings (prone knee flexion), quadriceps (seated leg extension), and abdominal muscles (curl-ups and prone planks).

In addition, a structured gait and balance training program tailored to the functional abilities of children with spastic diplegic cerebral was implemented. Each training session commenced with a 5 min warm-up (e.g., marching in place, dynamic stretching) and ended with a 5 min cool-down (e.g., breathing exercises, slow walking). The main gait and balance component lasted approximately 30 min per session and was conducted three times per week over a 12-week period. The training emphasized dynamic balance through various tasks and surfaces, progressing in complexity over time (Table 2). Each exercise was performed for 20 repetitions per set, with 3 to 5 sets depending on the child’s tolerance and fatigue level, allowing rest intervals of 30 to 60 s between sets. Progression was individualized, increasing task difficulty by altering the surface type, adding cognitive tasks, or reducing external support as motor control improved. Exercises were adapted according to the Gross Motor Function Classification System levels II–III [29].

### 2.5. Statistical Analysis

All statistical analyses were conducted using IBM SPSS Statistics for Windows, Version 27.0 (IBM Corp., Armonk, NY, USA). The Shapiro–Wilk test was used to assess the normality of the data distribution. Descriptive statistics were calculated for all variables, and independent (unpaired) *t*-tests were used to compare baseline characteristics (gender, age, height, and weight) between the two groups. A two-way analysis of variance with interaction effects was performed to evaluate differences between pre- and post-treatment measures for terminal stance, loading response, and initial contact phases in knee flexion, ankle plantar flexion, and ankle dorsiflexion, as well as outcomes on the Gross Motor Function Measure (GMFM) and Pediatric Balance Scale between the groups. A significance level of *p* < 0.05 was considered statistically significant for all tests.

## 3. Results

### 3.1. General Characteristics

**Figure 1:**
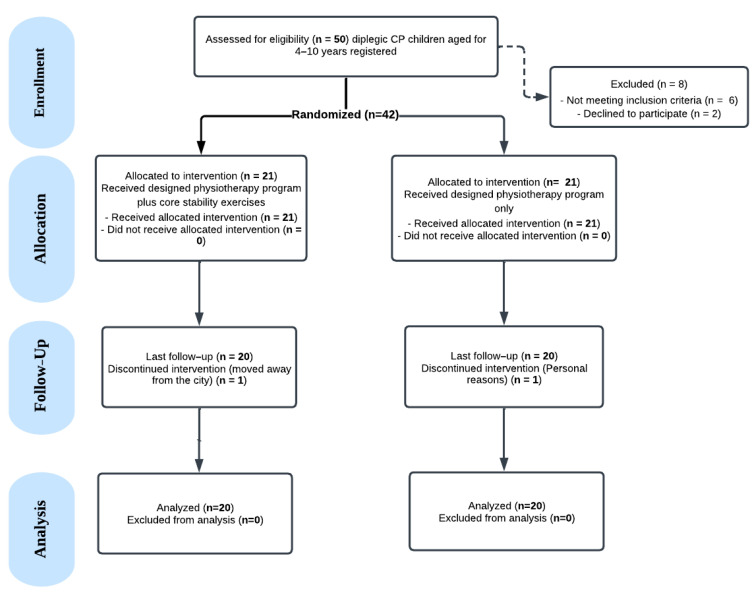
CONSORT flow chart for patients in the study.

The CONSORT flow diagram is shown in Figure 1. Fifty children were initially assessed for their eligibility. Eight participants were excluded: six did not meet the inclusion criteria and two declined to participate, resulting in 42 children enrolled in the study. Participants were randomly allocated into two groups (*n* = 21 each): the intervention group received core stability exercises in addition to a structured physiotherapy program, whereas the control group received the physiotherapy program alone. By the end of the intervention period, 40 participants had completed the study, and 2 participants were lost to follow-up.

Descriptive statistics for age, height, and weight revealed no statistically significant differences between the study and control groups at the baseline (Table 3). Some participants had a history of perinatal complications, including premature birth, anoxia (oxygen deprivation), and low birth weight. Others experienced cesarean delivery, labor involving suction procedures that resulted in cerebral cortex damage, prolonged stays in neonatal intensive care units following asphyxia, or severe neonatal jaundice.

### 3.2. Gross Motor Function Measures and Pediatric Balance Scale

**Figure 2:**
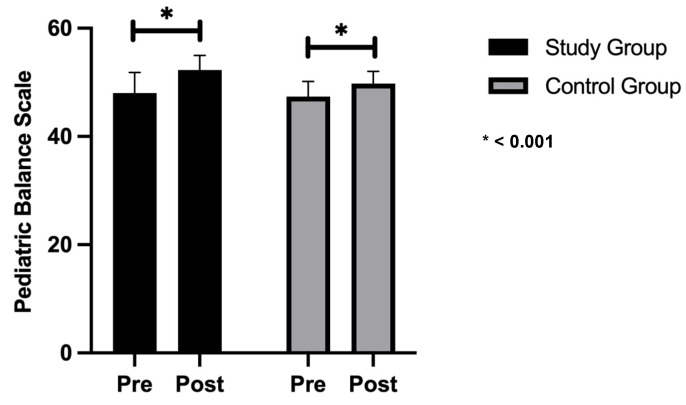
Comparison of Pediatric Balance Scale values in both groups.

**Figure 3:**
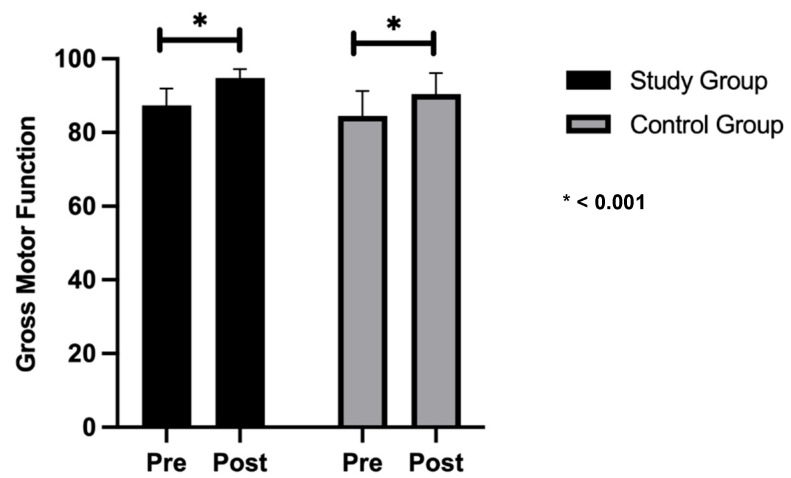
Comparison of mean values of Gross Motor Function Measure in both groups.

There was no significant difference in pre-treatment measures between the study and control groups, with mean values of 87.39 ± 4.59, 48 ± 3.83 in the study group and 84.56 ± 6.74, 47.4 ± 2.8 in the control group (*p* = 0.287, 0.694) for the Gross Motor Function Measure and Pediatric Balance Scale, respectively. Following the 12-week intervention, Figure 2 and Figure 3 illustrate that both the study and control groups demonstrated significant improvements. In the study group, the Gross Motor Function Measure and Pediatric Balance Scale improvements were 9%, whereas in the control group, they were 7% and 5%, respectively, indicating a significant difference between the groups in favor of the study group (*p* < 0.05), as shown in Table 4.

### 3.3. Knee Flexion During the Gait Cycle

**Figure 4:**
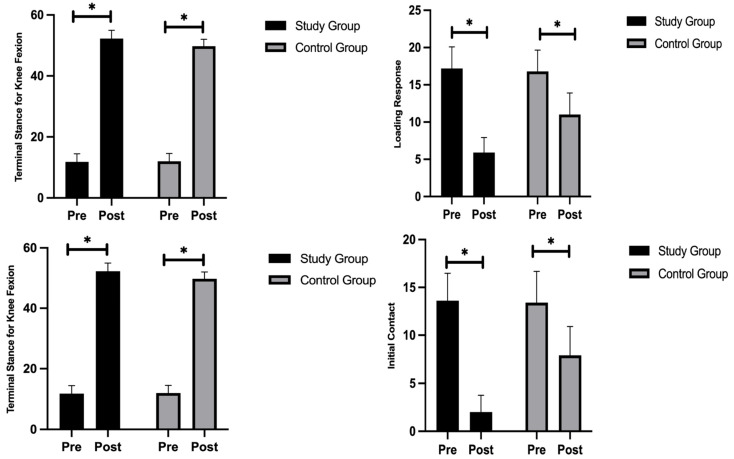
Comparison of mean values of gait parameters (* < 0.001).

Terminal stance is a standard phase of gait used to assess improvements in knee flexion. Before treatment, no significant difference was observed between the two groups, with mean knee flexion values of 11.8 ± 2.66° in the study group and 12 ± 2.58° in the control group (*p* = 0.866). The findings are presented in Figure 4. Both groups exhibited significant improvements after the intervention, with the study group showing an 81% increase and the control group showing a 41% increase (*p* < 0.05) (Table 5).

### 3.4. Ankle Plantar Flexion and Dorsiflexion During the Gait Cycle

The typical gait phase used to assess improvements in plantar flexion is loading response; the pre-treatment plantar flexion did not differ significantly between the two groups, with mean values of 17.2 ± 2.94 in the study group and 16.8 ± 2.86 in the control group (*p* = 0.761). Both groups exhibited significant improvements after the intervention, with the study group showing a 66% increase and the control group showing a 35% increase (*p* < 0.05). Both groups showed no significant difference in the pre-treatment dorsiflexion, and initial contact is the typical gait phase to assess dorsiflexion improvements, with mean values of 13.6 ± 2.88 in the study group and 13.4 ± 3.27 in the control group (*p* = 0.886). Both groups exhibited significant improvements after the intervention, with an 85% increase in the study group and a 41% increase in the control group (*p* < 0.05).

## 4. Discussion

This study compared the effects of core stability exercises, and a physiotherapy program designed for children with diplegic CP. The findings demonstrated statistically significant improvements in gross motor function, gait, and functional balance, as assessed by the Gross Motor Function Measure (GMFM), Kinovea software, and the Pediatric Balance Scale. Notably, core stability exercises produced superior outcomes across these measures. Children aged 4–10 years were selected based on the assumption that this age group is capable of participating in strength-training exercises, which are integral to core stability programs. This assumption was supported by Fry et al. [30], who reported the benefits of strength training in children aged three–seven. Furthermore, the results align with those of a previous study that found that trunk and pelvic training enhanced postural and equilibrium responses in children with CP [31]. Accordingly, core stability exercises appear to play a key role in improving postural control and balance in children with spastic CP.

It has been reported that a core stability program enhances trunk stabilization, which in turn improves the length–tension relationships of muscles in both the upper and lower extremities that originate from the girdles and connect to the spine [32]. This improvement also facilitates better phasic contraction of the spinal muscles, reduces freezing episodes, and increases degrees of freedom. Consequently, the movement becomes more appropriate, coordinated, and purposeful. Consequently, enhanced postural control may underlie the significant improvements observed in standing, walking, and balance during the early stages of intervention. This finding aligns with Bahramizadeh [33], who emphasized that postural adjustment plays a crucial role in movement efficiency by providing a stable foundation for active limb movement.

The study group showed a 9% improvement in standing and functional balance versus the control group (7%, 5%). According to previous research, it has been reported that prolonged standing using a standing frame led to positive effects on both balance and gait in young children with spastic diplegic CP. Proper positioning and plane adjustment in the device are important for maximizing benefits [34].

The superior outcomes of core stability exercises in improving knee flexion during the terminal stance—an 81% improvement compared to 41% in the control group—can be attributed to their positive impact on lower extremity muscle strength. Core stability enhances strength in both the upper and lower limbs. A more stable core allows for greater force production by the limbs [35]. Conversely, reduced core stability has been linked to increased knee flexion during the stance phase [36]. Somayeh [37] reported that core stability training on both stable and unstable surfaces improved ankle proprioception, dorsiflexion range of motion, and peak torque in both dorsiflexion and plantar flexion. These findings support the results, which showed an 85% improvement in dorsiflexion at initial contact and a 66% improvement in plantar flexion during the loading response following core stability exercises, compared with 41% and 35%, respectively, in the control group. Limitations in the ankle range of motion may alter the sagittal and coronal plane kinematics of the knee joint. Additionally, changes in foot and ankle posture may affect pelvic tilt, thereby influencing the overall posture of the trunk and lower limbs [38]. Despite the demonstrated benefits of core stability exercises for managing CP, research in this area remains limited. This scarcity of evidence presents a barrier to their widespread integration into therapeutic protocols despite their potential to improve gross motor function and balance. Expanding research in this field is essential to support their clinical adoption.

This study had several limitations that should be acknowledged. First, the wide age range of participants (4–10 years) may encompass varying stages of motor and cognitive development. Although functional classification (GMFCS levels I–II) and cognitive ability to follow instructions were used to ensure baseline comparability, developmental variability may still influence outcomes. Additionally, the gender distribution was not balanced, with more males than females enrolled. Although no significant sex-related differences were observed at baseline, this imbalance may affect the generalizability of the findings. Second, the study focused solely on children diagnosed with diplegic cerebral palsy and did not include participants with other types of CP, such as hemiplegic or quadriplegic CP. Therefore, the results cannot be generalized to a broader CP population. Additionally, all participants were classified as either level I or level II on the Gross Motor Function Classification System (GMFCS), representing children with relatively higher motor abilities. Consequently, the effects of core stability exercises on children with more severe motor impairments (GMFCS levels III–V) remain unclear. The narrow GMFCS range may have limited the detection of the effectiveness of the intervention across the full spectrum of motor function severity. Furthermore, there is an absence of General Movements Assessment data during the neonatal period. Although GMA is a valuable tool for early detection of neurodevelopmental disorders, it was not included in the analysis. This limits our ability to correlate early spontaneous motor patterns with later functional outcomes. Moreover, this study did not assess long-term outcomes, and follow-up evaluations were not conducted to determine whether the observed improvements were sustained over time. The lack of blinding and relatively small sample size also pose potential biases and limit the strength of the conclusions. Future studies should consider including a wider age range, different CP subtypes, and more diverse GMFCS levels. Additionally, the lack of a standardized assessment of the child’s motivation or emotional engagement could have influenced the effectiveness of core stability training. Given the known impact of motivation on adherence and performance in pediatric rehabilitation, future studies should consider incorporating validated motivation assessment tools to better understand its role in therapeutic outcomes.

## 5. Conclusions

This study demonstrated that both core stabilization training and conventional physiotherapy significantly improved gross motor function, postural control, and balance in children with cerebral palsy. The integration of core exercises led to better gait patterns, enabling children to achieve upright standing, move independently without recurrent falls, and perform dynamic movements, such as running across varied surfaces. These findings underscore the value of incorporating core stability training as a complementary therapeutic strategy in pediatric rehabilitation programs for children with CP. Importantly, these results contribute to the growing body of evidence supporting multimodal interventions to enhance functional mobility in this population. Future research is recommended to explore the long-term sustainability of these benefits and determine the effectiveness of home-based core stability programs in accelerating improvements in balance and gait.

## Figures and Tables

**Table 1:** Intervention protocol.

Frequency & Duration	3 sessions per week, 60 min per session
Session Structure	- 30 min conventional physiotherapy - 30 min structured core stability training
Conventional Physiotherapy (30 min)	- Postural control in kneeling, stepping, single-leg stance - Stretching: hip flexors, adductors, hamstrings, quadriceps, calf muscles (20 s hold × 3–5 reps) - Strengthening: hip abductors (side-lying), hip extension (prone hip extension), hamstrings (prone knee flexion), quadriceps (seated leg extension), abdominals (curl-ups, prone planks)
Core Stability Training (30 min)	Progressive levels (3 levels)
Level 1 (Beginner)	- Abdominal draw-in (20 reps) - Abdominal draw-in + double knee-to-chest (10–20 reps) - Supine trunk twists (10–20 reps)
Level 2 (Intermediate)	- Supine trunk bridging (3–5 reps) - Supine medicine ball twists (10–20 reps)
Level 3 (Advanced)	- Trunk bridging with back supported on physio-ball (hold 3–5 s × 10–20 reps) - Prone bridging (plank) (3–5 reps)
Rest Periods	30–60 s between exercises
Repetition of Sequence	Full sequence repeated 3–5 times per session based on tolerance

**Table 2:** Gait and balance training dosage.

Exercise	Description	Dosage	Progression
Walking on variable surfaces	Walking on soft mats, foam pads, and firm ground	20 steps per surface × 3 sets	Increase surface difficulty and reduce support
Transitioning between kneeling and standing	Practicing transitions from kneeling to standing	20 transitions × 3–5 sets	Increase repetitions or reduce assistive support
Step training (ascending and descending)	Using a 15 cm step platform to practice ascending and descending steps with support as needed	10 ascents + 10 descents per set × 3–5 sets	Increase step height or reduce assistance
Dual-task standing and walking activities	Walking while performing cognitive tasks such as holding an object or counting aloud	10 m of dual-task walking × 3 repetitions	Add more complex cognitive tasks or increase distance
Single-limb stance (eyes open and closed)	Balancing on one leg with eyes open or closed, near a stable support for safety	10–20 s per leg × 3 repetitions	Increase duration or progress from eyes open to closed

**Table 3:** General characteristics of both study and control groups.

Variable	Study Group	Control Group	*t*-Value	*p*-Value
	Mean ± SD	Mean ± SD		
Age (years)	6.2 ± 1.87	5.4 ± 0.97	1.2	0.246
Height (cm)	123.1 ± 6.67	121.9 ± 16.24	0.216	0.831
Weight (kg)	35.1 ± 7.13	28 ± 9.24	1.925	0.07

Note: SD, standard deviation.

**Table 4:** Comparison between and within the groups in the values of Gross Motor Function Measure and Pediatric Balance Scale in both groups.

Variables		Group		Mean Difference	*p*-Value Group × Time Interaction
		Study	Control		
		Mean ± SD	Mean ± SD		
Gross Motor Function Measure	Pre-test	87.39 ± 4.59	84.56 ± 6.74	2.83	0.29
	Post-test	94.82 ± 2.42	90.48 ± 5.67	4.34	0.3
	*p*-value	<0.001	<0.001		
Pediatric Balance Scale	Pre-test	48 ± 3.83	47.4 ± 2.8	0.6	0.69
	Post-test	52.3 ± 2.67	49.8 ± 2.2	2.5	0.04
	*p*-value	<0.001	<0.001		

**Table 5:** Comparison between and within the group in the gait parameters.

Variables		Group		Mean Difference	*p*-Value Group × Time Interaction
		Study	Control		
		Mean ± SD	Mean ± SD		
Terminal Stance	Pre-test	11.8 ± 2.66	12 ± 2.58	0.2	0.866
	Post-test	2.3 ± 1.7	7.1 ± 2.6	4.8	0.0001
	*p*-value	<0.001	<0.001		
Loading Response	Pre-test	17.2 ± 2.94	16.8 ± 2.86	0.4	0.76
	Post-test	5.9 ± 2.02	11 ± 2.91	5.1	0.0002
	*p*-value	<0.001	<0.001		
Initial Contact	Pre-test	13.6 ± 2.88	13.4 ± 3.27	0.2	0.88
	Post-test	2 ± 1.76	7.9 ± 3	5.9	<0.001
	*p*-value	<0.001	<0.001		

## Data Availability

None of the data obtained and produced in the scope of this study have been deposited in a publicly available repository, and the data will be made available on request.

## Abbreviations

The following abbreviations are used in this manuscript:

CP	Cerebral palsy
RCT	Randomized controlled trial
MAS	Modified Ashworth Scale
GMFCS	Gross Motor Function Classification System
PBS	Pediatric Balance Scale
